# Some Points on Tonsillitis

**Published:** 1889-02

**Authors:** C. Carleton Smith


### SOME POINTS ON TONSILLITIS.

It is always well to note the objective symptoms when prescribing for tonsillitis, for this reason: The patient may complain of aggravation from anything coming in contact with the throat. And on the strength of this symptom we may be led to prescribe Lachesis, but on this single indication alone we could not proceed with safety, for the reason that Apis-mel. and Kali-bich. each have the same symptom in their pathogenesis. Now, if we examine the tonsils and find one or both of them looking like transparent sacks filled with water, and the neck of the patient sensitive to pressure, Apis would be the remedy. On the other hand, if we find the fauces and tonsils covered with a tenacious mucus which the sufferer vainly tries to get rid of by hawking, on account of its sticky, ropy nature, Kali-bich. will be the remedy. When Merc-iod. is indicated, we will find the *root* of the tongue thickly covered with a *bright-yellow* deposit, the breath extremely fetid, and in place of tenacious mucus decided ptyalism will be present, with tonsils ulcerated and deglutition impossible. Lachesis has feeling as of a fish-bone sticking in throat. Hepar a sensation as of a splinter. When Lachesis patient swallows, a sharp pain shoots up into the ear of affected side, while with Kali-bich. the pain shoots from ear down into throat. The Lachesis patient fears he will choke to death when attempting to swallow, all fluids taken returning through the nostrils. This latter symptom is similar to Belladonna, but not in such a marked degree, I think, as under the former remedy. It is easy to choose, however, between the two with regard to this symptom, as under Bell. the patient's face is very red and hot, with some sweat, while under Lach. he is pale and anxious looking, with blue rings around the eyes, and there is no moisture. Lach. has aggravation from hot drinks; Lyc. aggravation from cold drinks; the former acting on the left side, the latter on the right.

Baryta-carb. will be found useful in those cases which are troubled with chronic enlargement of the tonsils, and when the attacks of quinsy tend speedily to suppuration, and where scrofulosis is a prominent factor. Hepar is the best remedy of the two, when this tendency to speedy suppuration obtains, if the patient complains of feeling acutely every little draft of air, and hence constantly calls out to have the doors kept shut. A very safe guide for the exhibition of Merc-sol. is perspiration quite profuse during the night of a sour odor, but which brings no relief; the saliva pouring from the corner of the mouth, wetting the pillow. The Phytolacca patient complains of intense dryness of the throat, so intensely dry that he feels it is impossible for him to swallow, though if he accomplishes it after persistent effort, he is rewarded by the most excruciating pains shooting up into both ears. It must not be forgotten that both Lach. and Kali-bich. have aggravation after sleep; but under the former remedy the patient sleeps *into* the aggravation, which awakens him each time to renewed suffering, while, under the latter remedy, the patient is worse after he gets his full quota of sleep.

Cases of tonsillitis that are tardy in clearing up, especially where suppuration has taken place, Sulphur is the proper remedy.

The tonsils should never be lanced, for such a procedure simply multiplies the attacks in the individual until he becomes subject to them both summer and winter. The law of similars is always equal to the emergency in these cases, provided the remedy selected is adapted to the individual case in hand, accordingly as the disease affects said individual case, always bearing in mind that there is no remedy for tonsillitis, but that there is a remedy for each and every individual suffering with tonsillitis, and that remedy will bring to him speedy and permanent relief.

C. Carleton Smith.

